# Gas-Purged Headspace Liquid Phase Microextraction System for Determination of Volatile and Semivolatile Analytes

**DOI:** 10.1155/2012/709656

**Published:** 2012-01-22

**Authors:** Meihua Zhang, Jinhu Bi, Cui Yang, Donghao Li, Xiangfan Piao

**Affiliations:** Key Laboratory of Nature Resource of the Changbai Mountain and Functional Molecular, Yanbian University, Ministry of Education, Park Road 977, Jilin Province, Yanji City 133002, China

## Abstract

In order to achieve rapid, automatic, and efficient extraction for trace chemicals from samples, a system of gas-purged headspace liquid phase microextraction (GP-HS-LPME) has been researched and developed based on the original HS-LPME technique. In this system, semiconductor condenser and heater, whose refrigerating and heating temperatures were controlled by microcontroller, were designed to cool the extraction solvent and to heat the sample, respectively. Besides, inert gas, whose gas flow rate was adjusted by mass flow controller, was continuously introduced into and discharged from the system. Under optimized parameters, extraction experiments were performed, respectively, using GP-HS-LPME system and original HS-LPME technique for enriching volatile and semivolatile target compounds from the same kind of sample of 15 PAHs standard mixture. GC-MS analysis results for the two experiments indicated that a higher enrichment factor was obtained from GP-HS-LPME. The enrichment results demonstrate that GP-HS-LPME system is potential in determination of volatile and semivolatile analytes from various kinds of samples.

## 1. Introduction

As is known, sample treatment is a very important stage of any analytical procedure. However, it takes much time to prepare the samples. It is reported that up to 80% of the total time is spent in preparing samples in a complete sample analysis process. So as to shorten time of the whole process of sample analysis, there is a trend towards integration and automation in modern sample treatment techniques. Among various sample treatment techniques, headspace liquid phase microextraction (HS-LPME), which integrates extraction, cleanup, and concentration, is a highly integrated sample treatment technique developed in recent years. In this technique, target compounds are evaporated from the sample matrix into the gas phase and then enriched by the solvent microdrop hanging on the tip of the microsyringe needle [1–3]. As a result of its characteristics of integration, simplicity, and rapidness, HS-LPME technique has been widely used for enriching volatile and semivolatile analytes from various kinds of sample matrixes [4–9].

For HS-LPME technique, the temperatures of sample matrix and extraction solvent are key factors that affect enrichment effect. Generally, a high temperature promotes release of target compounds from the sample and accelerates the diffusion of them into the gas phase, which speeds up the entry of target compounds into extraction solvent. Moreover, a low temperature of extraction solvent is beneficial to enrichment, because a low temperature is advantageous for target compounds in the headspace gas phase to dissolve in extraction solvent since the extraction of target compounds into the extraction solvent is an exothermic process [10–13]. Furthermore, in HS-LPME technique, the volume of headspace gas phase is limited because HS-LPME is performed in a closed system. Based on the theory of ideal gases, it is concluded that the amount of chemicals in the gas phase increases with increasing gas volume under a given temperature and pressure [14], so the amount of target compounds entering the headspace in HS-LPME is constant due to limited gas phase volume.

In practice, in order to obtain a high temperature of sample matrix and a low temperature of extraction solvent, various kinds of heating and cooling methods have been proposed. Recently, main heating methods such as hydrodistillation method, microwave heating, and recycling hot water have been used to increase the temperature of sample matrix. In addition, circulating cold water, CO_2_ cooling technique, and thermoelectric cooler have been used to cool the extraction solvent [15–17]. However, these methods have shortcomings including large device volume, inconvenience of operation, and difficulty in controlling the temperature and achieving online enrichment. Besides, much electrical energy is consumed due to use of microwave heating.

To achieve rapid, automatic, and efficient extraction, a gas purge headspace liquid phase microextraction (GP-HS-LPME) system was researched and developed here, in which semiconductor condenser and heater were designed, respectively, to cool the extraction solvent and to heat the sample (the ranges of refrigerating and heating temperatures are from −5°C to room temperature and from room temperature to 125°C, resp.). Besides, inert gas was constantly led into and discharged from the system for the purpose of accelerating the movement of target compounds and increasing the gas phase volume in GP-HS-LPME system by prolonging extraction time. Thus, the amount of target compounds in the headspace gas phase is raised and the enrichment factor is improved. In order to evaluate the enrichment ability of this system, extraction experiments were performed with 15 polycyclic aromatic hydrocarbons (PAHs) standard mixture using original HS-LPME technique and GP-HS-LPME system, respectively, and the extraction solvents were analyzed by GC-MS. The results indicate that a higher enrichment factor can be obtained by GP-HS-LPME system compared with the one obtained by original HS-LPME technique. Moreover, the GP-HS-LPME system represents advantages of simplicity of the operation, automation, and accuracy of the control in extraction conditions, and rapidness of the extraction.

## 2. Experimental

### 2.1. Fabrication of the Semiconductor Condenser and Heater in GP-HS-LPME System

**Figure 1:**
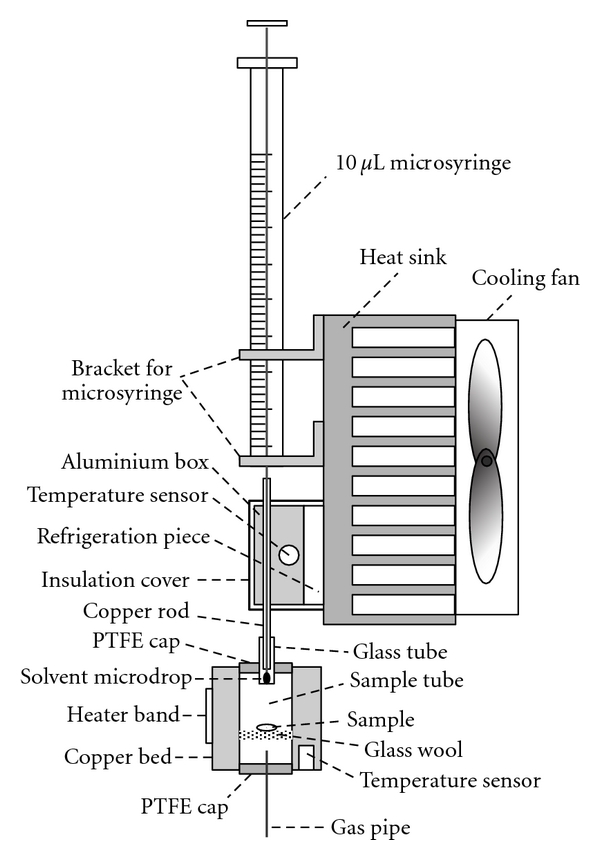
Schematic of the semiconductor condenser and heater in GP-HS-LPME system.

A semiconductor condenser used for GP-HS-LPME, which was an application of Peltier effect [18, 19] of semiconductor to refrigeration, was constructed according to the principle of thermoelectric refrigeration [20]. As is shown in the upper part of Figure 1, the semiconductor condenser is composed of the aluminum box, temperature sensor, copper rod, refrigeration piece, insulation cover, heat sink, cooling fan, and the brackets for microsyringe.

As is shown in Figure 1, the hot side of the refrigeration piece was attached to the heat sink and cooling fan combination to dissipate the generated heat. An aluminum box inside which a column was machined to fix a hollow copper rod (external and internal diameter of the copper rod are 1.4 mm and 0.5 mm, resp.) was attached to the cold side of the refrigeration piece. In order to detect the current temperature of the condenser, a hole was machined at the side of the aluminum box to embed the temperature sensor. To protect the extraction process from the influence of environmental temperature, a perspex insulation cover was mounted covering the aluminum box and refrigeration piece and it was fixed tightly to the heat sink by screws. Two small holes were machined separately in the upper and lower parts of the insulation cover to pass through the copper rod. On one side of the heat sink, brackets for microsyringe were mounted to hold the microsyringe at a proper settled height. After passing the microsyringe needle continuously through the brackets and the hollow copper rod, the microsyringe was finally stabilized at the brackets.

To increase the temperature of sample matrix, a metal-oxide ceramic heater (MCH) was designed in GP-HS-LPME system. Besides, for enhancing enrichment factor, inert gas was introduced into the system. As is shown in the lower part of Figure 1, the heater consists of the copper bed, heater band, two PTFE caps, glass tube, sample tube, glass wool layer, temperature sensor, and the gas pipe. A cylinder was machined inside the copper bed for placing the sample tube and a glass wool layer on which sample was placed was set inside the sample tube. Two PTFE caps were used to cover the top and bottom of the sample tube, respectively. A glass tube (external and internal diameter are 3.7 mm and 1.8 mm, resp.), which was inserted into the top PTFE cap to serve as the gas outlet channel for the inert gas, made the GP-HS-LPME system become an open system. A gas pipe bringing in the inert gas was inserted into the sample tube by sticking it in the bottom PTFE cap. The gas pipe was connected to the mass flow controller (not shown in Figure 1), which was used for measuring and adjusting the gas flow of inert gas led into the system. To monitor the current temperature of the heater, a temperature sensor was embedded in the copper bed. A MCH heater band was attached on the surface of the copper bed so as to generate heat to heat the sample tube when electric current was applied to it.

In order to achieve a good cooling effect of the extraction solvent, the copper rod described above in the condenser was plunged into the glass tube (gas outlet channel) of the heater, which was close to the microsyringe needle during the extraction process.

### 2.2. Constitution and Working Principle of GP-HS-LPME System

**Figure 2:**
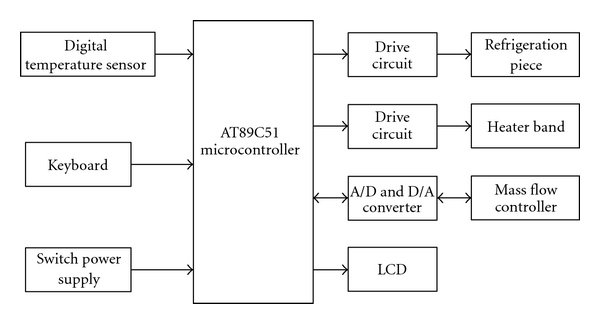
Electrical schematic diagram of GP-HS-LPME system.

The GP-HS-LPME system is mainly composed of the semiconductor condenser, heater, switch power supply, microcontroller, keyboard, and LCD. Switch power supply is employed for supplying power to the system, keyboard is equipped to set refrigerating temperature of the semiconductor condenser and to set the heating temperature of the heater and gas flow rate of inert gas introduced, and LCD is used to display the above parameter values accordingly.  Figure 2 illustrates the electrical schematic diagram of the total system.

The semiconductor condenser consists of AT89C51 microcontroller [21], DS18B20 1-wire digital temperature sensor [22], FPH1-3120NC semiconductor refrigeration piece, drive circuit for the refrigeration piece, switch power supply, LCM141 LCD module [23], and 4 × 4 keyboard. The heater is composed of AT89C51 microcontroller, DS18B20 temperature sensor, MCH heater band, drive circuit for the heater band, PCF8591 8-bit A/D and D/A converter [24], S49-32B/MT mass flow controller [25], switch power supply, LCM141, and 4 × 4 keyboard. 

After setting refrigerating and heating temperature, gas flow rate of inert gas and timing time by keyboard and starting the GP-HS-LPME system, by control of the microcontroller, the whole system can work automatically according to the set value and the working process is as follows. 

**Figure 3:**
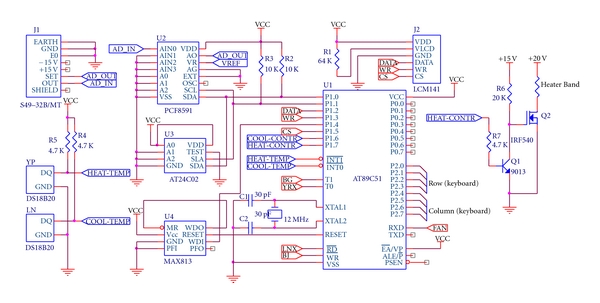
Control circuit diagram of GP-HS-LPME system.

Figure 3 illustrates the control circuit diagram of GP-HS-LPME system. First, two DS18B20 temperature sensors are used to measure the current temperatures of the semiconductor condenser and the heater, respectively; they convert the two temperatures directly into two digital electric signals with 12-bit reading each (for each DS18B20, default 12-bit resolution is adopted and the converted thermal data is stored in the scratchpad memory in a 16-bit, sign-extended two's complement format, sign bits indicate whether the temperature is positive or negative). Next, the two digital signals are transferred, respectively, over the 1-wire interface of DS18B20 to AT89C51 microcontroller by issuing Read Scratchpad [BEh] commands when the temperature conversions have been performed. Then, the microcontroller calculates error amounts by comparing the temperatures measured by two DS18B20 sensors with the ones set by keyboard, and PID algorithm is employed to figure out controlled variables. Two PWM (Pulse Width Modulation) signals are generated according to the controlled variables and are used to drive the refrigeration piece and MCH heater band to work by connecting the PWM signals to drive circuits for the refrigeration piece and the heater band, respectively. If the measured temperature value by DS12B20 sensor does not correspond to the set value, the microsyringe needle and sample tube will be cooled and heated by the refrigeration piece and MCH heater band, respectively, and ultimately reach and retain the set value until the timing time is over. 

At the same time, S49-32B/MT mass flow controller (MFC) is adopted in GP-HS-LPME system to measure and control the gas flow of inert gas introduced into the sample tube through the gas pipe. The detected gas flow analog signal by MFC is transferred to a piece of PCF8591 to be A/D-converted to 8-bit digital signal, and then the signal is input into AT89C51 microcontroller through I^2^C interface. The microcontroller calculates error amount by comparing the measured gas flow rate with the one set by keyboard and PID algorithm is adopted to figure out controlled variable, which is converted into digital control signal and exported by microcontroller. To be input into drive circuit for gas flow solenoid valve (inside the mass flow controller), the exported digital control signal should be connected to the piece of PCF8591 to be converted into analog control signal, which will be connected to the drive circuit and drive the gas flow solenoid valve to turn up or turn down so as to adjust gas flow of the inert gas. Thus, inert gas introduced into the system will finally reach and remain the set gas flow rate until the timing time is over. 

In conclusion, through the control of the AT89C51 microcontroller, the whole system can achieve the required refrigerating and heating temperature and gas flow of inert gas rapidly and automatically.

### 2.3. Experimental Procedure

To perform an extraction experiment using GP-HS-LPME system, the operation process is as follows. (1) The real or standard sample was put on the glass wool layer inside the sample tube; the sample tube was put into the copper bed and covered by two PTFE caps both on the top and the bottom. (2) The glass tube was inserted into the top PTFE cap and the copper rod was plunged into the glass tube. The gas pipe, which brings in inert gas, was inserted through the bottom PTFE cap. (3) Suitable extraction solvent was added into the microsyringe, and then the microsyringe was inserted continuously through the brackets for microsyringe and the copper rod into the glass tube. (4) The microsyringe plunger was depressed and solvent microdrop formed on the tip of the microsyringe needle, and the height of glass tube was adjusted so as to make the microdrop of extraction solvent locate where it just fills the glass tube. (5) The system was applied to set suitable values of gas flow rate, refrigerating and heating temperatures and timing time, and then the extraction started. (6) After the set extraction time is time out, the solvent microdrop was retracted back to the microsyringe and the microsyringe was removed from the system. Finally, the extraction solvent within the microsyringe was injected to the GC-MS for composition analysis. 

## 3. Results and Discussion

### 3.1. Optimization of GP-HS-LPME

The gas-purged headspace liquid phase microextraction system was applied in the determination of volatile and semivolatile chemicals. The phenanthrene, anthracene, fluoranthene, and pyrene were used as typical chemicals. In order to obtain high enrichment efficiency for volatile and semivolatile analytes from various kinds of samples, the parameters that affect enrichment factor in GP-HS-LPME system, such as the gas flow rate, the position of the extraction solvent microdrop, the diameter of the glass tube, the temperatures of the extraction solvent and the sample, and the extraction time, were systematically optimized. The optimized conditions were 2.7 mL min^−1^ for the gas flow rate of the inert gas, the extraction solvent microdrop filling the glass tube, 1.8 mm for the internal diameter of the glass tube, −6°C and 80°C for the temperatures of the extraction solvent and of the sample, respectively, and 20 min for the extraction time. Of the various parameters, higher temperature of the sample and lower temperature of the extraction solvent are greatly favorable for high enrichment factor and reducibility of the GF-HS-LPME technique as reported by Yang et al. in 2009 [14]; the desirable values of the parameters are easily obtained and accurately controlled using semiconductor condenser and a metal-oxide ceramic heater developed here. 

### 3.2. Evaluation of GP-HS-LPME System

Under the optimization conditions, 15 PAHs standard mixture samples were extracted using the GP-HS-LPME system for evaluation of enrichment factor and reproducibility of the technique. In addition, contrastive experiment was done under the identical optimized parameters for the same kind of sample using the HS-LPME technique. The extraction solvent chosen for the two extraction experiments was dodecane and the amount of extraction solvent used for each was controlled at 2 *μ*L for the subsequent GC-MS analysis. In this study, the 15 PAHs standard mixture was also used to evaluate the reproducibility of GP-HS-LPME system and original HS-LPME technique. The reproducibility was represented by the relative standard deviation (RSD). It can be seen from Table 1 that enrichment efficiency of GP-HS-LPME was enhanced by 4 times higher than this of HS-LPME, and the RSD value of the GP-HS-LPME system ranged from 4.56% to 9.45% and this value ranged from 8.96% to 19.38% for the HS-LPME technique, which demonstrated that GP-HS-LPME revealed better reproducibility. 

### 3.3. Sensitivity of GP-HS-LPME for Volatile and Semivolatile

**Figure 4:**
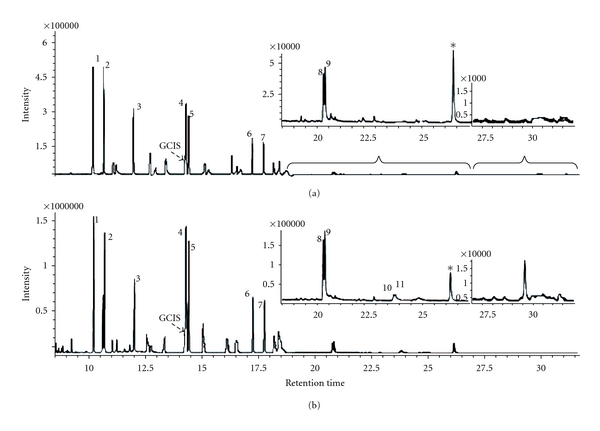
Chromatograms of target compounds enriched by HS-LPME (a) and GP-HS-LPME (b) 1: Acenaphthylene; 2: Acenaphthene; 3: Fluorene; 4: Phenanthrene; 5: Anthracene; 6: Fluoranthene; 7: Pyrene; 8: Benzo[a]fluoranthene; 9: Chrysene; 10: Benzo[b]fluoranthene; 11: Benzo[k]fluoranthene.

In order to evaluate sensitivity of the GP-HS-LPME system on the volatile and semivolatile chemicals, the 15 PAHs standard mixture which covered wide range of boiling point (from 298.9°C to 561.1°C at 760 mmHg) was used as target chemicals, and the results were compared with this obtained by the HS-LPME. As is shown in Figures 4(a) and 4(b) denote the chromatograms of target compounds analyzed by GC-MS after using HS-LPME and GP-HS-LPME technique. Total of 11 and 9 compounds were, respectively, detected by the GP-HS-LPME and HS-LPME. In Figure 4, Numbers 1–11 indicate 11 kinds of target compounds enriched by the two kinds of extraction techniques. Although 1–9 compounds were detected from both GP-HS-LPME and HS-LPME, the intensity obtained by the GP-HS-LPME was about 3-4 times as high as those in the HS-LPME. Furthermore, 10-11 compounds were hardly found in the HS-LPME case, while they were easily found in the GP-HS-LPME case. Comparing the two intensity values of each target compound enriched by HS-LPME and GP-HS-LPME, it could be concluded that higher enrichment efficiency could be obtained by using GP-HS-LPME system developed here for determination of volatile and semivolatile chemicals in contrast with the usage of HS-LPME technique.

## 4. Conclusion

With the use of semiconductor condenser and heater, the volume of the system was reduced and the stability was improved. The advantages of simplicity of operation, automatic control of experimental parameters (conditions), and high efficiency of extraction enable GP-HS-LPME system to be used in enriching both volatile and semivolatile target compounds from various kinds of samples. The enrichment factor of the GP-HS-LPME is 3 or 4 times as high as this value of HS-LPME. In addition, because of its ability of low-voltage power supply (e.g., a car battery) and miniaturization of the device, GP-HS-LPME system enables on-site and online field enrichment of analytes from different samples. Thus, GP-HS-LPME system has a wide prospect that was applied in the enrichment of different kinds of target compounds from various sample matrixes in fields such as food chemistry, biochemistry, and environmental chemistry.

## Figures and Tables

**Table 1:** Comparison of the GP-HS-LPME and HS-LPME.

Compound	GP-HS-LPME		HS-LPME		GP-HS-LPME/HS-LPME
	Analyte/GCIS	RSD (%)	Analyte/GCIS	RSD (%)	
AcPy	36.18	6.45	9.17	10.78	3.95
AcP	34.64	4.56	8.36	9.04	4.14
Flu	23.97	7.03	7.64	8.96	3.13
Phe	30.56	8.16	7.78	12.09	3.93
AnT	27.97	4.69	7.23	15.37	3.87
FluA	12.86	6.38	2.88	16.31	4.47
Pyr	11.93	5.03	2.74	17.83	4.35
B[a]F	2.17	9.45	0.57	19.03	3.81
Chr	2.52	8.53	0.62	19.38	4.06
B[b]F	0.35	6.81	—	—	—
B[k]F	0.28	5.39	—	—	—
B[a]P	—	—	—	—	—
IND	—	—	—	—	—
DBA	—	—	—	—	—
B[ghi]P	—	—	—	—	—

—undetection; AcPy: Acenaphthylene; AcP: Acenaphthene; Flu: Fluorene; Phe: Phenanthrene; AnT: Anthracene; FluA: Fluoranthene; Pyr: Pyrene; B[a]F: Benzo[a]fluoranthene; Chr: Chrysene; B[b]F: Benzo[b]fluoranthene; B[k]F: Benzo[k]fluoranthene; B[a]P: Benzo[a]pyrene; IND: Indeno(1,2,3-cd)pyrene; DBA: Dibenz(a,h)anthracene; B[ghi]P: Benzo(ghi)perylene.
